# Scaling up the global reef restoration activity: Avoiding ecological imperialism and ongoing colonialism

**DOI:** 10.1371/journal.pone.0250870

**Published:** 2021-05-06

**Authors:** Mark T. Gibbs, Bridget L. Gibbs, Maxine Newlands, Jordan Ivey

**Affiliations:** 1 Australian Institute of Marine Science, Townsville, Australia; 2 School of Earth and Environmental Sciences, The University of Queensland, Brisbane, Australia; 3 The Nature Conservancy, Brisbane, Australia; 4 College of Arts, Society & Education, James Cook University, Australia; 5 Bundjalung, South Sea Islander, Australia; 6 Faculty of Science and Environment, Southern Cross University, Lismore, Australia; Leibniz Centre for Tropical Marine Research, GERMANY

## Abstract

The health and condition of the world’s reefs are in steep decline. This has triggered the development of fledgling micro-scale coral reef restoration projects along many reef coastlines. However, it is increasingly recognised that the scale and productivity of micro-scale coral gardening projects will be insufficient to meet the growing global threats to reefs. More recently, efforts to develop and implement restoration techniques for application at regional scales have been pursued by research organisations. Coral reefs are mostly located in the unindustrialised world. Yet, most of the funding, and scientific and engineering method development for larger-scale methods will likely be sourced and created in the industrialised world. Therefore, the development of the emerging at-scale global reef restoration sector will inevitably involve the transfer of methods, approaches, finances, labour and skills from the industrialised world to the unindustrialised world. This opens the door to the industrialised world negatively impacting the unindustrialised world and, in some cases, First Nations peoples. In Western scientific parlance, ecological imperialism occurs when people from industrialised nations seek to recreate environments and ecosystems in unindustrialised nations that are familiar and comfortable to them. How a coral reef ’should’ look depends on one’s background and perspective. While predominately Western scientific approaches provide guidance on the ecological principles for reef restoration, these methods might not be applicable in every scenario in unindustrialised nations. Imposing such views on Indigenous coastal communities without the local technical and leadership resources to scale-up restoration of their reefs can lead to unwanted consequences. The objective of this paper is to introduce this real and emerging risk into the broader reef restoration discussion.

## Introduction and background

Most of the world’s coral reefs are located in unindustrialised nations, many of which are located in the Southern Hemisphere, and the global decline of coral reef condition is now well-documented [1]. Few reefs have escaped exposure from local threats in the form of over-exploitation, catchment runoff and direct habitat destruction and global threats in the form of increasing regional ocean temperatures and changes to the acidity of ocean waters [2]. Coral reefs in unindustrialised nations are global hotspots of biodiversity and integral to the livelihoods of coastal communities through the provision of protein from fisheries. Many of these coastal communities are First Nations communities [3].

To date, the management of coral reefs in the industrialised world has focused on managing anthropogenic activities undertaken directly on, or adjacent to, reefs [4]. Commonly used policy instruments seek to address managing fishing effort (often through spatial zoning), or more recently through managing activities undertaken on adjoining reef catchments such as runoff from agricultural practices [5, 6]. Such management approaches do not manage reefs themselves, but rather the human activities that impact reefs [7]. These management and policy instruments can be effective when the dominant stressors on reefs are essentially local [8]. By contrast, the most effective way to deal with global changes such as anthropogenic climate change, which are essentially external and hence beyond the control of local reef management agencies and organisations, is to reduce the hazard on a global scale [9]. In the case of ocean warming and acidification, this implies reducing global greenhouse gas emissions [10]. This is the course of action advocated by a number of prominent members of the reef research community [6]. Whilst making logical sense, there are two substantial challenges to this course of action which limit the effectiveness with regard to minimising bleaching on coral reefs in the tropical oceans.

Firstly, local reef managers, as well as the global reef research and advocacy community, are not well placed to directly control, diffuse and mitigate worldwide global emissions. In many cases, local reef managers struggle to control activities occurring in their own reef catchments [6]. Secondly, the inertia in the climate system is such that by the time global emissions are finally harnessed and contained, coral reefs will be so degraded that recovery will be challenging if not impossible [11]. Many argue that a number of reef systems are already in this condition [12]. Furthermore, management of reefs in unindustrialised nations is generally based more on customs and traditional management practices than the administrative and technocratic approaches typically used in industrialised nations. Therefore, management practices developed and implemented in the industrialised world often do not easily translate to the unindustrialised world [13].

Given this situation in both the industrialised and unindustrialised world, it is understandable that a number of communities, researchers and reef stakeholders have begun trials for restoring reefs (a summary of these current practices can be found in [14]). The majority of restoration activity in both the industrialised and unindustrialised world has focused on coral gardening approaches whereby fragments of corals are raised in in-situ nursery areas through attaching fragments to artificial support structures, then out-planting these fragments back onto reefs [15]. These approaches are typically micro-scale (restoration scales on the order of tens of square metres), costly (tens to hundreds of thousands of dollars to restore a few hundreds of square metres of reefs [15]), and labour intensive. These current practices are commonly undertaken by local communities, often in the Small Island Nations or unindustrialised coastal nations [for example 16], and often under the direction of non-government organisations (NGOs) and research organisations based in industrialised nations–despite the fact that most reefs are geographically located in unindustrialised nations. Many of these current practices aspire to scale up to the order of hectares and in some cases larger. However, the lower bound estimate of the total global area of coral reefs is 255 000 km^2^ [17], and recent bleaching events during the last decade have decimated thousands of square kilometres of reefs [12]. Operations at the scale of current practices (tens of square metres) will require exponential replication to achieve an increase in scale of many orders of magnitude in order to demonstrably reverse the current trajectory of many, if not most reef systems [18].

Achieving such a scale will require substantial investment in method research and development (such as the Australian Government Reef Restoration and Adaptation Program [RRAP]) [19], the construction and operation of infrastructure and technology and the deployment of skilled labour resources, much of which is not currently accessible (e.g., in the Small Island Nations). This will all need to be underpinned by sustained funding. However, despite the fact that the majority of the world’s reefs are located in unindustrialised nations (often the Small Island Nations), many if not most of the attributes and capabilities identified above are commonly sourced from industrialised nations. Therefore, it is likely that at-scale reef restoration will require the transfer of know-how, technology and biological materials such as corals from industrialised nations to unindustrialised nations. Similarly, community-based coral gardening typically involves collecting fragments from corals, rearing them and out-planting onto reefs close to the source of the fragments. In such cases, the coral community and genetic structure among reefs is relativity unchanged due to the small geographic distance between the source of the fragments and the out-planted corals [15]. By contrast, as more and more areas of reefs become unviable, techniques such as assisted gene flow and translocation [20] of corals from more distant geographic areas will be required, which opens up the requirement for more advanced research to be undertaken. Decisions must be made regarding what species and genetic traits are to be replanted, and more importantly, who gets to make these choices. As restoration techniques become more technically complicated, the ability of the Small Island Nations or other Indigenous communities to undertake restoration activities by themselves decreases as a result of the lack of technical capability and capacity within these communities. Many of these nations will need to be supported by industrialised nations which host these capabilities.

This can bring forth a one-way flow of knowledge and organic materials, including potentially corals, that is often forced upon Indigenous communities and First Nations peoples, be it deliberate or undeliberate, leading to a phenomenon known in Western academia as ecological imperialism. This phenomenon has been occurring for centuries. In the nineteenth century, there was a dramatic increase in ecological imperialism, with a similar transfer of knowledge and materials to that of today. This left many nations with drastically altered landscapes and biodiversity and has led to the ongoing social and cultural impacts on First Nations peoples [21, 22]. Therefore, the risks and potential unintended consequences of global reef restoration activities, akin to colonialism in general and ecological imperialism specifically, need to be not only explicitly considered in the development of the emerging at-scale global reef restoration sector but also when grappling with the greater issue of how Western science interacts with traditional management practices when it comes to at-scale reef restoration.

## The Western concept of ecological imperialism

Colonialism was practiced by the ancient empires that expanded and subsequently contracted over many centuries. The expansion of the Roman Empire and establishment of Viking strongholds across northern Europe are well-known examples. These colonisations are often celebrated as leading to the spread of architecture, language and culture, often to the detriment of remembrance of the violence inflicted upon colonised peoples. Modern Colonialism commenced in the late 15^th^ century as Europeans began to invade the American continent in earnest, with the first colony established in North America in Virginia in 1607.

Between1820 and 1930, over 50 million Europeans colonised a relatively small number of countries including the United States, Canada, Australia, New Zealand, and South American countries such as Argentina and Uruguay [23]. This represented a migration of around 20% of the global population at the time, exceeded in numbers only by the recent migration (last 50 years) occurring within almost every continent from inland rural regions to urbanised cities, often located on the coast [24]. This more recent nineteenth-century colonisation involved relocations of people from temperate Europe to other counties with mostly, but not exclusively, temperate climates. From the perspective of colonising neo-Europeans, the ‘new’ lands often featured seasonal climates similar to the climate in their ‘home’ countries, but settlers found the local flora and fauna to be often dramatically different. For example, in what is now known as Australia, colonisers found kangaroos where they might have expected cattle. The authors all live in Australia and hence are most familiar with examples from this continent, and now living on land and sea country that is dramatically different to pre-colonisation. Colonisers accustomed to deciduous trees found trees that retained their leaves year-round [25] and sought to ‘fix’ these ecosystems. By contrast, traditional owners of the land saw this process as an invasion whereby the invaders not only occupied their land and sea country but set out to actively re-model their traditional ecosystems.

For some, ecological imperialism is a modern form of colonialism in which the populations in postcolonial eras continue to have an immense impact on unindustrialised countries [26]. For First Nations peoples, the very idea of what is described/conceptualized as ecological imperialism is abrasive and offensive. This nineteenth-century colonisation by Europeans extended mainly across the tropics and torrid zone [27]. With this colonialism came the induction of small-scale industrialisation, urbanisation and disease, leading to concentration of populations, increasing coastal and catchment development and associated impacts on ecosystems, traditional cultures and the very existence of many First Nations peoples [28, 29].

Colonisers justified the persistent push to import the familiar plants and animals from the homeland given the unfamiliarity of newly colonised landscapes and climates, as well as their lack of knowledge of how to harness the local flora and fauna for food, fibre, clothes and transportation. Colonisers proactively sought to make their new home like their old home based on their Western concept of what a ‘natural’ landscape should look like. English house sparrows (*Passer domesticus*) were introduced to North America by the nostalgic Englishman Nicolas Pike in 1850, and camels were introduced to outback Australia during the same period [30]. New Zealand became the home of outdoor European sport wildlife with deer, game birds, trout and salmon being introduced [31]. Cattle and sheep were widely introduced and distributed across the new nation. ‘Acclimatisation’ societies developed in response to this new demand. In the UK, the Society for the Acclimatisation of Animals, Birds, Fishes, Insects and Vegetables (established in 1860) enjoyed strong and sustained support from naturalists and philanthropists from English society who sought to Europeanise distant colonies [32]. In Australia, the similarly named and tremendously influential Queensland Acclimatisation Society commenced in 1862 and was operating up until 1956 to introduce new crops to the colonies [33, 34]. However, this rural industry development caused the significant flow-on effect of biodiversity loss. In Australia alone, twenty species of mammals have been declared extinct, and nearly half of marsupial and monotreme species are now on the extinct, endangered or vulnerable list as a result of habitat loss and degradation since European settlement. Currently, on average, 100 million reptiles are lost year on year from land clearing alone in Australia, much of which is still associated with agriculture of non-native species [35]. It can also be argued that the way communities interact and utilise flora and fauna in Australia remains to be in the context of Anglo-Saxon traditions.

From the perspective of First Nations peoples, the colonisers not only invaded but actively remodelled the landscapes and biodiversity, and for cultures that are deeply connected to land and sea country, the colonisers impacted the very core of their culture. In Australia, the impacts on Aboriginal and Torres Strait Islander peoples from these ecological regime shifts have been profound [36]. Australian historians frame this as a ‘logic of elimination’, where colonisers reduce and redefine Indigenous peoples to a problem to then justify removing them from the land [37]. For those communities allowed to stay on country (their ancestral lands), it is not often the same country of their ancestors. Many Indigenous Australians still experience lower levels of employment and education, higher levels of morbidity and mortality in response to both mental and physical health, lower life expectancy, and higher incarceration rates when compared to non-Indigenous people [38]. The ongoing intergenerational trauma of racism continues to disadvantage First Australians, and this is particularly noticeable when considering the dominant methods of communicating and performing science in Australia. These are all considered to be flow-on impacts attributable to ecological imperialism and colonisation in general [39]. The legacy of colonisation and ecological imperialism activities occurring over a century ago continue to have ongoing consequences and impacts on many continents and islands, including Australia, which still appear to be more pronounced for First Nations peoples.

### Ecological imperialism and Western conservation approaches

Predominantly Western scientific approaches imposed on non-Western cultures tend to lack acknowledgment of the intrinsic social cultural value attributed to species and social practices such as land rights and tenure systems [40]. Furthermore, it can be argued that undertaking token attempts to incorporate traditional ecological knowledge into Western-style decision-making processes can lead to even more detrimental outcomes than taking no consideration at all. Relevant to this discussion, reef restoration projects need an awareness of socio-cultural practices if they are to avoid ecological imperialistic attributes. For example, it has been argued that the existing narrative for conservation management in the Coral Triangle needs to move away from Western-scientific preservationist values and towards utilitarian values, putting importance on local food security, traditional practices and human development priorities [41]. Similarly, in the Solomon Islands, certain coral species are bestowed totemic status, making them taboo for particular groups of peoples that go above and beyond their ecological values [42].

Rather than remaining a bygone process of previous centuries, ecological imperialism is still actively occurring and, in some cases, increasing with globalisation. For example, references to ecological imperialism can be commonly found in the climate change mitigation and adaptation literature with regard to the allocation of past and future greenhouse emissions [43]. Similarly, the research literature identifies ecological imperialism effects occurring in emerging states where fossil fuels have been discovered by oil and gas multinational corporations [44]. Ecological imperialism is often enshrouded in larger concepts such as the ‘neoliberalization of nature’, which encompasses how natural capital is another form of capital that becomes concentrated under neo-liberal policies [45]. By contrast, to date, there has been a paucity of consideration of the emerging risk of ecological imperialism occurring in the domain of reef restoration. This is somewhat concerning given the opportunity for ecological imperialism to be enacted as the global at-scale reef restoration sector amplifies.

## Why is reef restoration at risk of ecological imperialism and ongoing colonialism?

The risk for ecological imperialism occurring in the context of the development of global reef restoration activity can be explored by considering the risk pathways through which colonisation in general and ecological imperialism in particular might creep into restoration activities. These are discussed below.

### Risk pathway 1: Biological and ecological methods

At the heart of any Western science coral restoration methodology is the biological and ecological restoration target outcomes, with biological outcomes often framed in Western ecological language and concepts and which focuses on coral (growth/survivorship, thermal/acidification tolerance and genetic diversity) or ecological services (coral diversity, fish biomass and diversity, benthic diversity, recruitment and structural complexity). Methods to deliver these outcomes might include asexual and sexual methods of propagation and managing corals at a range of life-stages from larvae to reproducing adults [14]. Method development is expected to be an ongoing activity as methods are developed, refined and scaled up. The majority of reef restoration research programs have focused on developing new methods and testing them on micro-scales [15]. These approaches are Western-science centric. As highlighted above, a key challenge of current practices is, therefore, to scale up these existing methods to regional scales, at least. In order to achieve this up-scaling, decisions need to be made regarding what species to restore, and often implicitly, what reef ecological goods and services will be maximised. For example, reefs can be rapidly restored by installing metal or concrete structures as new substrate and then planting fast growing Acropora corals onto this new artificial substrate [46]. This approach is targeted towards rapid restoration of coral cover by utilising non-reef materials (e.g., metal rebar and concrete) in reef environments but comes at the cost of reducing coral resilience to disease, predation and bleaching (unless the planted corals are thermally tolerant), due to a lack of genetic and species diversity. Other choices include using only naturally occurring reef products for substrate modification and focusing on maintaining diversity by restoring slower-growing species, which stretches out the timelines and increases the cost of restoration. These choices are essentially social and economic decisions and best made by local stakeholders to avoid ecological imperialism and ultimately exclude Western aspirations about what a reef ‘should’ look like and prevent the project’s purpose (i.e., tourism or sustainable fishery) from being superimposed on reefs. By contrast, the outcomes for restoration projects identified above are intrinsically ecology centric rather than human centric. This is a critical point, as for many Small Island Nations, the largest source of financial revenue is from international tourism facilities that are often owned and developed by industrialised nation corporations [47]. One can imagine a scenario whereby ‘designer’ reefs, possibly configured around Disney characters are designed in restoration projects to please international tourists in order to generate revenue for multinational corporations at the possible expense of a protein source for local communities. Alternatively, local stakeholders may choose to embark on a rapid single-species restoration strategy in order to maintain a sub-set of ecosystem services (e.g., coastal protection) that sacrifices biodiversity and perhaps longer-term resilience values for shorter-term needs. However, such a program may well be inconsistent with published ecological restoration design principles as advocated by the largely Western restoration ecology community [48].

### Risk pathway 2: Deployment technology and infrastructure

There are two fundamental approaches to upscaling the global reef restoration activity. The first approach involves replicating micro-scale operations such as coral gardening over and over. This can be thought of as the ‘domestic’ development model of restoration. This is how the global agricultural sector developed over almost the entire history of human civilisation–simply applying more and more labour to more and more land using the same small-scale methods [49]. By contrast, during the latter half of the twentieth century, changes to farming following the widespread introduction of machinery, new breeds, pesticides and nutrients, often applied using broadacre (including aerial) methods, hailed the introduction of industrial agriculture [50]. This so-called green revolution led to dramatic changes in per hectare and per worker productivity and yield, especially in industrialised nations [51]. Applying this approach can be considered the ‘industrial’ approach to reef restoration; pioneered in the industrialised world despite the well-understood negative impacts of industrial agriculture, which include pesticide and nutrient runoff to coral reefs [52]. The industrial upscaling approach is reliant upon new technology to be developed to assist in underwater and surface (including on-deck) operations, as well as on-land technology (possibly including genetic technology) in land-based aquaculture facilities. However, if this technology is expensive and requires highly skilled labour to operate and maintain, then once again we are in ecological imperialism territory with a boomerang effect. Put simply, the boomerang effect [53] is when a wealthier country opens up a business in unindustrialised nations, access to which by the local community is restricted. The workers are often from the same country as the parent company (see risk pathway 3 below) and will often spend income earned back home rather than ‘in country’ in the local community. For example, in the global oil and gas sector, teams of highly skilled contractors from industrialised nations fly in and undertake on-the-ground activities to international, rather than local, specifications using highly sophisticated technologies, and then fly out. This creates an economic bubble that is only beneficial to the industrialised nation and often leaves the recipient unindustrialised nation in a worse state through, for example, the ‘Dutch’ disease of oil and gas development [44]. It is conceivable that a technologically developed workforce sourced from an industrialised nation could undertake fly-in, fly-out reef restoration activities in unindustrialised nations with little or no local community direct involvement. This approach can be especially infuriating for First Nations communities. There can be a clear trade-off between ecological outcomes and local stakeholder outcomes. Pertinent other examples of existing industries are the global salmon farming industry, which is underpinned largely by Norwegian marine farming support industries and technology [54] and the global mining industry that is supported by infrastructure and technologies from the industrialised world, including Australia [55].

### Risk pathway 3: Non-local labour force

Reef restoration activities are undertaken by people. Therefore, scaling is dependent not only on access to technology but also access to labour to apply the technology. In the micro-scale replication or domestic model of upscaling, labour skill requirements, and hence productivity, is generally low. For example, the labour productivity of current methods of reef restoration is estimated to be at least two orders of magnitude less than current industrial practices in agriculture [18]. Therefore, this domestic approach to upscaling is very labour intensive. However, technology-based industrial approaches can require a more-upskilled labour force which may be challenging for First Nations communities. Working with local people together with technological approaches in shared knowledge and co-design can help mitigate ecological imperialism risk. Therefore, both the capacity and capability of the local labour force is a critical determinant for upscaling. Furthermore, many coral reef systems are located in remote coastal locations where local labour can be sparse. However, many of these reefs provide key cultural values and ecosystem services to communities that extend beyond their geographical location. Maintaining these essential ecosystems services may require restoration of remote reefs which are not located close to available labour sources, and this opens up the opportunity for non-local labour with little ‘skin in the game’ to be required for restoration.

### Risk pathway 4: Financing

Reef restoration requires labour, technology and deployment infrastructure. The mobilisation of these resources requires financing. At present, many reef restoration programs are funded through international aid programs or through existing tourism operations that are financially supported through the industrialised world [56]. With funding comes influence, and the source of funding for reef restoration in unindustrialised nations can be a large potential source and risk pathway for ecological imperialism. In addition, many funding agencies, including philanthropy sources, are often hesitant to allocate funds to organisations in unindustrialised nations that do not have a ‘corporate’ or Western governance structure [57]. This forces many small and emerging organisations in unindustrialised nations to take on Western governance structures as well as corporate project planning and delivery cultures in order to attract funding, forcing local organisations to ‘Westernise’, which is also another form of colonisation.

The four ecological imperialism risk pathways identified above are enabled by the Western concept of social licence to operate (SLO), which in itself is a concept that can be abhorrent to First Nations peoples. SLO is commonly viewed as a way to reduce business risk by gaining social acceptance from local and regional communities [58]. The origins of SLO are often attributed to the development of corporate social responsibility initiatives in the industrialised world in response to the demise of the reputations of especially major corporations operating in the territories of Indigenous peoples [59]. In the early 2000s, the United Nations identified a requirement that industries secure free, prior, and informed consent (FPIC) from Indigenous peoples [59]. SLO reflects a classic colonisation notion that ‘permission and acceptance’ equals justification for an action [60]. As highlighted above, colonisation occurs when non-local influences are allowed to undertake in-country activities without gaining local stakeholder consent.

It has been argued that the very concept of social licence is a Western concept that is not well understood or practiced in many unindustrialised nations; despite the fact that the concept was developed to allow foreign-owned companies to operate in unindustrialised nations [61]. Therefore, insisting on pursuing the concept of gaining social licence can be abhorrent to First Nations peoples and establishing social licences in unindustrialised worlds can be problematic as the traditional form of consensus decision-making often differs from the form of decision-making practiced in democratic industrialised nations. Many unindustrialised countries do not rigidly follow conventional Western power structures, but instead tend to govern themselves with a combination of conventional Western power structures and cultural practices [61]. Of the 33 countries in the world that have the Westminster system of government (an upper and lower house of governance), at least 17 rely on coral reefs for sustainability [62]. For example, in Papua New Guinea the political and regulatory regime operates under a Westminster government arrangement (a hangover from colonisation), but there is also the Wantok cultural system of kinship to take into account. Similarly, Samoa’s legislative system combines the Westminster and chiefly systems [63]. Therefore, whilst social licencing may dovetail well in Western legislative systems, applying Western power structures to an unindustrialised country is also a form of ecological imperialism when ecosystems are altered.

Many of the negative consequences of colonisation and ecological imperialism influence what is commonly known as the Global South. The Global South is defined by the Brandt line as an economic zone that splits the world into developed, developing and least developed countries [64]. Moreover, the difference between Global North and South can be traced back to the ‘exploitation of the South during the colonial period and has continued since the end of the European empires’ [65]. For example, in Pacific Island Nations environmental problems are increasingly linked to economic development, as these nations strive for more economic security. However, this entails the risk of a catch 22 when unindustrialised countries rely on/become reliant on external assistance from wealthier countries that are prone to imparting ecological imperialism [66].

Therefore, taking an approach that includes social licence but excludes cultural relativism runs a high risk of losing trust amongst stakeholders and, in many cases, is in principle unethical. This reinforces the risk pathways whereby there is lack of alignment in terms of the desired restored biological assemblage or community structure, associated trade-offs of ecosystems goods and services delivered by the restored reef system (both of which are Western ecological constructs), and traditional cultural norms and values.

### Implications of risk pathways

All four of the attributes identified above (methods, people, equipment & technology, and finance) are essential for at-scale restoration to be achieved. However, of relevance to this discussion, the framework these pathways are operated within (e.g., Western research, aid and NGO organisations in the context of SLO) will largely determine the scale of ecological imperialism effects and ongoing colonisation. The logic of this is as follows:

For the domestic model of upscaling:

- Current practices of reef restoration follow the domestic development model. These are largely funded by international NGOs and larger individual tourism operators who are often owned by non-local organisations.
- The NGOs (often based in industrialised nations) generally seek funding from industrialised-world philanthropists, and larger tourism operations also seek visitors from wealthy nations.
- NGOs also commonly utilise volunteer labour from industrialised nations for reef restoration operations.

For the industrialised model of upscaling (yet to be developed):

- These industrialised approaches are in the very early phase of development (for example [19, 67]). However, it is clear from these early works that such approaches will require the development of new technologies and methods that are mostly being developed in industrialised nations but will be required to be applied in unindustrialised nations.

No matter which development pathway is pursued, if Western-developed reef restoration practices are to be globalised than they will create both the demand for restored reefs (through tourism), and the supply of restoration methods and services to unindustrialised nations. In other words, a transfer of demand, resources and knowledge from industrialised to unindustrialised nations is likely to occur. This is, therefore, the vector through which ecological imperialism, and ongoing colonialism, can be activated. This is also precisely the mechanism of ecological imperialism through colonisation during the 1800s during which demand, technology, labour and financing were transferred from the industrialising nations to the unindustrialised world. Human culture and landscapes of the new world replicated the old world to the ongoing detriment of First Nations peoples. There is a current risk ecological imperialism will occur again with the scaling up of reef restoration, in which industrialised nations, knowing or unknowingly, will pressure First Nations peoples to develop designer reefs and reef communities that replicate the attributes and expectation of ecological and human communities in those industrialised nations. By creating a new activity, and possibly an industrialised reef restoration support sector in unindustrialised nations, there is a real risk of Westernisation of not only the underwater ecological communities but also the human reef communities, including First Nations peoples, which practitioners of reef restoration should be aware of and strive to avoid. Below, we outline a number of suggestions on how this may be achieved.

## Summary and concluding remarks

One of the major bottlenecks in gaining consensus for global greenhouse gas emissions targets has been the challenge of working in the face of injustices whereby unindustrialised nations understand that the old-world industrial collective of nations has both created the anthropogenic climate change problem and has also contributed more than its fair share of the global atmospheric carbon budget [43], working in light of the global inequity of the consequences of emissions. Coral reefs are front and centre in these arguments, as the majority of coral reefs are located in the unindustrialised world. Yet, the major stressor to these reefs is primarily a result of the industrialisation of the already industrialised world. Transferring capital, methods, labour resources and technology developed in the industrialised world to unindustrialised nations that host coral reefs is a pathway for reparation from the impacts of global emissions. However, this mechanism can also be used as a Trojan horse for further colonisation through the vector of ecological imperialism, whereby assistance leads to undesired and unwanted social and ecological impacts on reefs and reef human communities in unindustrialised nations. Industrialised nations may provide assistance to the unindustrialised world in order to facilitate opportunities for research providers and commercial organisations to capitalise on opportunities in unindustrialised nations and create ecosystems that suit the interests of industrialised nations.

From a First Nations perspective, even clinically identifying and labelling perceived past misdemeanors as ecological imperialism can be abrasive, as if by labelling we are consigning it to past misbehaviours. However, we have clearly not learned as we are embarking upon a new crusade to develop and impose Western-science-developed knowledge in approaches to reef restoration onto the unindustrialised world, where most coral reefs are located. For example, the first question to consider in any reef restoration program is often, ‘What should the attributes of the restored reef be?’. However, in order to answer this, we must have an understanding of, at minimum, how local stakeholders value reefs and how they should participate in the design, management and implementation of at-scale activities. This is particularly the case when restoration may become a major community activity and source of employment. Whilst seemingly straightforward, given the complexities of likely research and development and implementation supply chains for capital, labour and technology, the management of these issues can be problematic. This is especially the case for small communities such as in the Small Island Nations. With regards to this first question above, the scientific (mostly Western) community has developed ecological design principles for reef restoration. However, as argued here, these may not necessarily reflect the view of individual communities, and Western science is only beginning to learn how to find a pathway through the parallel, but largely independent, if not mutually exclusive, approaches to Western and Indigenous resource management.

Fortunately, this is not a new problem. Over recent decades, foreign governments and increasingly the international NGO sector have been ramping up ‘in country’ activities to work with local stakeholders to achieve development and sustainability outcomes. It is now recognised that many of these earlier aid-type programs were essentially following the colonisation pathway–seeking to nudge unindustrialised world communities and settlements into Westernised versions [68]. For example, funding the replacement of housing and accommodation structures made of non-permanent traditional materials with structures made of permanent materials (that are often less resilient to natural hazards) in cyclone-exposed Pacific Islands [69].

More recent in-country activities undertaken by foreign governments and the global NGO sector are increasingly culturally sensitive and relevant. However, this approach is challenging and resource intensive. Anne Ross [70] described a number of systemic and epistemological barriers to integrating the knowledge of Indigenous communities into natural resource management. Systemic barriers reflect the influence of Western power structures that remain embedded in environmental management and practice [71]. Different knowledge systems should not be a barrier to Indigenous peoples having input into environmental decisions. Yet, systemic factors such as bureaucratic arrangements (e.g., meeting requirements) and government structures, as well as the decentralized nature of Indigenous concepts around governance and decision-making, make it challenging for Indigenous peoples to participate [72]. Systemic or institutional factors are also perpetuated by the State, which has more power than Indigenous people do, and are often focused on meeting global environmental conservation objectives rather than solving local resource challenges. Therefore, it can be argued that Indigenous peoples continue to be disadvantaged by colonial assumptions about the way inferior ‘native races’ use, or fail to use, natural resources [72].

Epistemological barriers often relate to the way that the knowledge of Indigenous communities is expressed. Barriers continue to arise when Western systems disregard the relevance of social, cultural and spiritual forms of Indigenous knowledge, and when Western property rights, including intellectual property rights, impose over Indigenous peoples’ rights [35]. The Westernised requirement to have information written down, or translated, poses another problem for Indigenous communities who have concerns over codifying and appropriating knowledge. Similarly, narrowly defined concepts of ‘tradition’ and ‘custom’, as well as management planning systems that require spatial and temporal bounds as marked on maps, continue to exclude Indigenous peoples from having a meaningful input. Sadly, Indigenous peoples’ traditional knowledge, expertise and connections to the land are in many cases yet to be validated by the majority of Western scientists and managers, and there is still a lack of recognition that Indigenous resource stewards once had some influence and impact on natural resource management despite many examples of how this can be achieved [73]. This will have to be addressed for reef restoration practitioners and projects to become attuned to local priorities instead of following external agendas.

In summary, global at-scale reef restoration is in its infancy, although micro-scale restoration efforts are underway. However, the preconditions for scaling up reef restoration activities, many of which will be led by industrialised nations, also create an environment whereby unintentional and unwanted ecological imperialism and ongoing colonisation may result. Therefore, a key message from this thought-piece is that organisations embarking on the at-scale restoration journey need to be aware of the possible consequences of ongoing colonialization through ecological imperialism and the potential for new colonialism. In its simplest form, managing this risk consists of recognizing when plans and discussions undertaken in research organisations in the industrialised work drift into ecological imperialism, especially through the risk pathways identified here. The next level of consideration includes actively involving not only local research organisations where they exist, especially local communities in unindustrialised nations in restoration projects, but empowering them to take the lead on specifics of the restoration outcomes sought, including which species to restore. In fact, non-local participants should consider (rather than try to fit local customs and approaches into Western-style analyses and decision approaches) to take the reverse approach by seeking methods to incorporate Western-style technocratic approaches into established Indigenous management systems. Increasingly Western research programs develop projects in partnership with local institutions. However, it can be argued that, in many cases, this consists of allowing local labour to participate in delivering Western research programs rather than viewing them as equal partners if not research and operations directors [74]. There is a difference between using local labour sources to deliver imperialistic outcomes in unindustrialised nations versus true partnerships with local stakeholders to deliver culturally sensitive restoration outcomes. This also represents a conundrum in that, as previously identified, detractors of restoration highlight that the productivity of current restoration practices precludes them from reversing the decline in global reef condition. Furthermore, the capacity and capability to develop methods that dramatically increase productivity primarily reside in industrialised nations and are not necessarily easily adapted to culturally aware implementation (and are additionally hindered by perceptions of inefficiency of non-Western approaches) in the nations where most reefs are located. Yet, aiming for culturally sensitive/locally attuned/locally led approaches to reef restoration is imperative in order to address the global coral reef crisis that disproportionately affects countries and communities in the Global South.
